# Outbreak of Cutaneous Leishmaniasis in Peruvian Military Personnel Undertaking Training Activities in the Amazon Basin, 2010

**DOI:** 10.4269/ajtmh.15-0107

**Published:** 2015-08-05

**Authors:** Marianela Oré, Eliana Sáenz, Rufino Cabrera, Juan F. Sanchez, Maxy B. De Los Santos, Carmen M. Lucas, Jorge H. Núñez, Kimberly A. Edgel, Justino Sopan, Jorge Fernández, Andres M. Carnero, G. Christian Baldeviano, Juan C. Arrasco, Paul C. F. Graf, Andres G. Lescano

**Affiliations:** Inteligencia Sanitaria, Comando de Salud del Ejército, Lima, Perú; Servicio de Dermatología, Hospital Militar Central, Lima, Perú; Dirección General de Epidemiología, Ministerio de Salud, Lima, Perú; Escuela de Medicina, Universidad Peruana de Ciencias Aplicadas, Lima, Perú; U.S. Naval Medical Research Unit No. 6 (NAMRU-6), Lima, Perú; Dirección de Salud Lima Sur, Ministerio de Salud del Perú, Lima, Perú; Universidad Peruana Cayetano Heredia, Lima, Perú

## Abstract

Military personnel deployed to the Amazon Basin are at high risk for cutaneous leishmaniasis (CL). We responded to an outbreak among Peruvian Army personnel returning from short-term training in the Amazon, conducting active case detection, lesion sample collection, and risk factor assessment. The attack rate was 25% (76/303); the incubation period was 2–36 weeks (median = 8). Most cases had one lesion (66%), primarily ulcerative (49%), and in the legs (57%). Real-time polymerase chain reaction (PCR) identified *Leishmania* (*Viannia*) *braziliensis* (59/61 = 97%) and *L.* (*V.*) *guyanensis* (2/61 = 3%). Being male (risk ratio [RR] = 4.01; *P* = 0.034), not wearing long-sleeve clothes (RR = 1.71; *P* = 0.005), and sleeping in open rooms (RR = 1.80; *P* = 0.009) were associated with CL. Sodium stibogluconate therapy had a 41% cure rate, less than previously reported in Peru (∼ 70%; *P* < 0.001). After emphasizing pre-deployment education and other basic prevention measures, trainees in the following year had lower incidence (1/278 = 0.4%; *P* < 0.001). Basic prevention can reduce CL risk in deployed militaries.

## Introduction

Leishmaniasis is a group of diseases caused by protozoal parasites of the genus *Leishmania* and transmitted by the bite of the female sand fly.^1^ Cutaneous leishmaniasis (CL) is the most frequent type of leishmaniasis, affecting 0.6–1.2 million persons each year around the globe.^2^ Its clinical features are varied, ranging from a mild, self-healing, localized lesion to severe, disseminated presentations.^3^ Despite continued control efforts, the incidence of CL has increased in the last decade in many regions of the world, including Latin America,^4^,^5^ where one-third of all cases worldwide are concentrated.^2^ Peru specifically is one of the 10 countries reporting 70–75% of the cases worldwide.^2^ CL is endemic in over 70% of the country's territory, primarily in the rainforest, and is most frequently caused by *Leishmania* (*Viannia*) *braziliensis*, *L*. (*V*.) *peruviana*, *L*. (*V*.) *guyanensis*, and *L*. (*V*.) *lainsoni*.^6^ In 2011, over 9,000 new cases were reported, representing an important increase since 2008.^7^

The recent spread of leishmaniasis has been attributed to human activities leading to the exposure of nonimmune populations to infection, including travel and migration, civil conflicts, and military operations.^3^,^4^,^8^,^9^ The armed forces are a particularly vulnerable population since they are an immunologically naive group often deployed to highly endemic areas for training or operational activities. Outbreaks of CL have been previously described in military populations deployed to endemic settings in Latin America, with attack rates ranging from < 1% to 91%.^10^–^17^

In the Andean and Amazonian regions of South America, the scale-up of military activities to combat illicit drug trafficking and terrorism in various endemic settings^18^ provides an avenue for the continued transmission of CL.^19^ In recent years, outbreaks among military personnel have been reported in Brazil,^14^–^17^ Colombia,^20^ the British Guiana,^21^ and the French Guiana.^10^,^22^ In particular, about 40,000 cases of CL were reported in Colombia between 2005 and 2009 in military personnel deployed to the jungle to fight against drug trafficking and guerrilla groups.^20^ Although no outbreaks have been reported among military personnel deployed to sylvatic areas in Peru, a retrospective study of 331 cases of leishmaniasis among military personnel treated at a major military hospital between 1997 and 2000 found that most of the affected people had been serving duty in the Peruvian Amazon Region, and had a soldier rank.^23^ These data suggest that greater military presence in endemic areas may result in a surge of CL transmission, and underscores the importance of studying the transmission, diagnosis, and management of the disease.

In this report, we describe the response to an outbreak of CL in military personnel deployed to the Peruvian rainforest for short-term training. Active case detection, close follow-up of the cohort, and a comprehensive questionnaire helped to understand the epidemiology of CL in these settings and identify modifiable risk factors strongly associated with increased risk. We then provided pre-deployment CL prevention education regarding the risk factors identified to the next trainees to be deployed and increased awareness among the chain of command, observing substantially lower CL incidence. We believe that this structured evaluation–action approach can help to prevent CL outbreaks among military personnel deployed to endemic areas.

## Materials and Methods

### Training activity.

**Figure 1.**
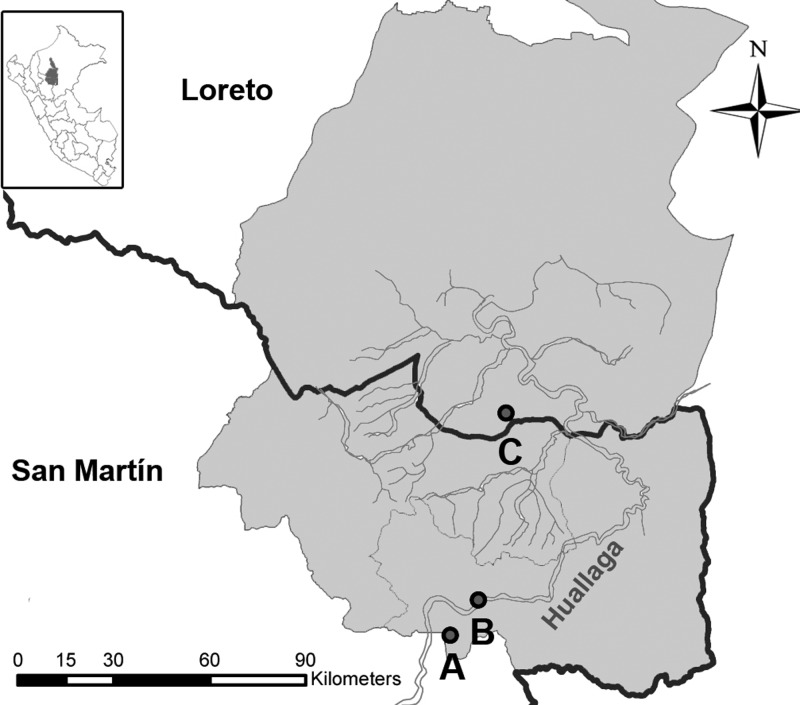
Map of Alto Amazonas in the northern Peruvian Amazon basin. Map shows the three sites (**A**, **B**, and **C**) where military personnel undertook short-term survival training. Dark lines show the border between the San Martin and Loreto departments and blue lines show river beds. Global positioning system (GPS) coordinates were intentionally removed for being considered sensitive information.

On January 30, 2010, second-year military personnel based in Lima, Peru were deployed to a 4-week survival training in the Alto Amazonas area, northwest Peruvian Amazon Basin (Departments of San Martin and Loreto, near the Huallaga River; Figure 1

). This region is endemic for leishmaniasis, malaria, and other infectious diseases.^24^ During the 4-week training, the temperature in the area varied from 19.9°C to 37.3°C and the total rainfall was 79.2 mm. After completing this training activity, all personnel returned to Lima without subsequent deployments to endemic areas.

### Outbreak investigation and response.

On May 5, 2010, the Peruvian Army Health Command (COSALE, for its acronym in Spanish) was notified cases of cutaneous lesions occurring in personnel that completed the aforementioned training. Physical examinations were scheduled at the Central Military Hospital (HMC, for its acronym in Spanish) in Lima on May 7 and a total of 52 potential cases were initially identified, suggesting the occurrence of an outbreak. COSALE then organized the outbreak response in fulfillment of their public health mandate within the Peruvian Army. COSALE requested epidemiological and diagnostic support from the General Epidemiology Directorate (DGE, for its acronym in Spanish) of the Peruvian Ministry of Health (MoH) and the U.S. Naval Medical Research Unit No. 6 (NAMRU-6). Initial microscopy testing was performed at HMC and the MoH Reference Laboratory of the Health Directorate II, south Lima. Diagnostic confirmation and identification of the infecting species was performed at NAMRU-6. Culture and molecular assays confirmed 52 first cases of leishmaniasis, and active case detection was initiated to identify potential additional cases, reaching out to all personnel who attended the training course. Physical examinations, collection of lesion tissue, and laboratory tests were planned and conducted from May 7 to 21, and after that ad hoc assessments were scheduled if military personnel started to present lesions. CL cases were defined as military service members deployed to the training area as part of the aforementioned training having one or more cutaneous lesions compatible with CL and with laboratory confirmation of the *Leishmania* parasite. Confirmed cases received supervised treatment and post-therapy follow-up by medical personnel of COSALE in strict compliance with the guidelines of the Peruvian MoH for first- and second-line therapy. In brief, intravenous sodium stibogluconate at 20 mg base Sb/kg/day for 20 days is one of the recommended first-line therapy for CL in Peru. Patients with treatment failure undergo an additional 20-day treatment course with a pentavalent antimonial at the standard dose. If cure is not achieved after the second course with a first-line agent, treatment is continued with amphotericin B at 0.5–1.0 mg/kg/day until remission or a cumulative dose of 1.5–2.0 g.^25^

### Data collection.

In-depth interviews were conducted among several of the first 52 cases and their training officers to identify and describe potential risks during training. A structured questionnaire was used to collect demographic, epidemiological, and clinical data. MoH epidemiological surveillance data and case reports from facilities near the training area were reviewed. All personnel that traveled to the training area underwent detailed physical examinations performed by dermatologists at HMC. Lesion samples were collected from all individuals with cutaneous lesions compatible with CL. Individuals who did not present lesions initially were followed up for 8 months post-deployment to monitor lesion appearance and were recommended not to travel to endemic areas during that period.

### Lesion samples.

Depending on the type, location, and size of the lesions, different types of samples were taken, including punch biopsies, lancet scrapings, and lesion aspirates to increase the chance of a positive diagnosis. Punch biopsies were taken from the border of the lesions using a 3-mm-diameter biopsy puncher and under local anesthesia with 2% xylocaine. The biopsies were then placed in 1.5-mL microfuge tubes containing 1 mL of 70% alcohol and in one case the sample was fixed with paraffin and stained with hematoxylin for histopathology. For lancet scrapings, lesion materials were superficially scraped using a scalpel and 2% xylocaine, and scraped material was placed into a vial containing 70% alcohol. Aspirate samples were obtained using a syringe with a 22-gauge needle containing isotonic saline (0.9%) with antibiotics and antifungals.

### Diagnostic methods.

Microscopy was performed at the MoH Reference Laboratory of the Health Directorate II and histopathology was performed at HMC. Culture and kinetoplastid DNA-based polymerase chain reaction (kDNA PCR) using the primers MP1-L and MP3-H were performed at the Department of Parasitology of NAMRU-6 as previously described.^6^,^26^ A novel real-time PCR assay that was previously developed and validated at NAMRU-6 was used to determine the species of *Leishmania*.^27^ This real-time PCR assay is based on fluorescence resonance energy transfer–based melting curve analysis of reactions targeting the mannose phosphate isomerase (MPI) and 6-phosphogluconate dehydrogenase (6PGD) genes.

### Post-outbreak surveillance.

Military personnel participating in similar survival training activities in the following year received leishmaniasis awareness briefings aimed at promoting the use of personnel protective measures to prevent sand fly bites following the findings of the risk factor assessment. After returning from training, personnel underwent physical examination to detect skin lesions using the same procedures and follow-up as during the outbreak.

### Statistical analysis.

The incubation period was estimated from the period with the highest likelihood of exposure until the epidemiological week of the first appearance of cutaneous lesions. The epidemic curve was prepared by counting cases in consecutive calendar periods defined by one-fourth of the approximate median incubation period.^28^ The main outcome of the epidemiological analyses was the attack rate, calculated as a cumulative incidence: the number of confirmed CL cases divided by the number of participants in the training (population at risk). Contingency tables were used to identify potential risk factors associated with an increased incidence of CL, and statistical significance was determined with chi-square^2^ or Fisher's exact test, as appropriate. A multiple Poisson regression analysis was used to determine the independent effect of each potential risk factor and estimate the risk ratio (RR) of CL after adjusting for potential confounders.^29^ This approach was preferred over traditional logistic regression because the attack rate in all exposure categories was substantially higher than 10% and the incidence odds ratio would overestimate the RR.^30^ Robust variance estimates were calculated to avoid distortion of confidence intervals. All tests were two-tailed and a 5% significance level was considered relevant. All statistical analyses were performed using Stata 11.0 for Windows (StataCorp LP, College Station, TX).

### Ethical considerations.

The outbreak response was conducted in compliance with the international ethical guidelines for biomedical research and all applicable federal and international regulations governing the protection of human subjects, particularly considering the vulnerability of military personnel. Outbreak responses are public health interventions conducted by authorized national authorities such as COSALE addressing their public health mandate, and therefore do not meet the requirements to be considered human subject research nor require review and approval of research ethics committees. Nevertheless, oral informed consent was obtained from all personnel, as traditionally done in outbreak investigations conducted by national public health authorities, and all samples and data were collected strictly for the public health response to the outbreak. Treatment was provided expeditiously in strict adherence to Peruvian national guidelines. The NAMRU-6 Institutional Review Board was also informed about the activities to be conducted before the outbreak response started.

## Results

A total of 303 military personnel were deployed to the survival training course. Most were male (89%), young adults (range = 18–23; average = 20.4 years), and born and raised in non-endemic areas (92%). None reported a history of leishmaniasis.

The in-depth interviews with the personnel and officers revealed that the course took place in three stages (Figure 1). First, personnel performed 3 weeks of exercises in site A, a military base located in a small rural area at 890 m above sea level (m.a.s.l.), where no CL cases had been reported by the MoH in 2010. Then, personnel completed a 24-hour, 12-mile hike through the rainforest arriving to site B (260 m.a.s.l.). During this hike, all personnel wore pants and long-sleeve shirts. Finally, they were transported in open vehicles to site C (148 m.a.s.l.), where they completed endurance training for 4 days. These activities included an open field exercise without long-sleeve clothing. Personnel were split into smaller groups based on their height, and all groups performed the same activities in parallel. Epidemiological and exposure data suggested that site C was the site at highest risk of infection and week 4 was used to estimate the incubation period.

**Figure 2.**
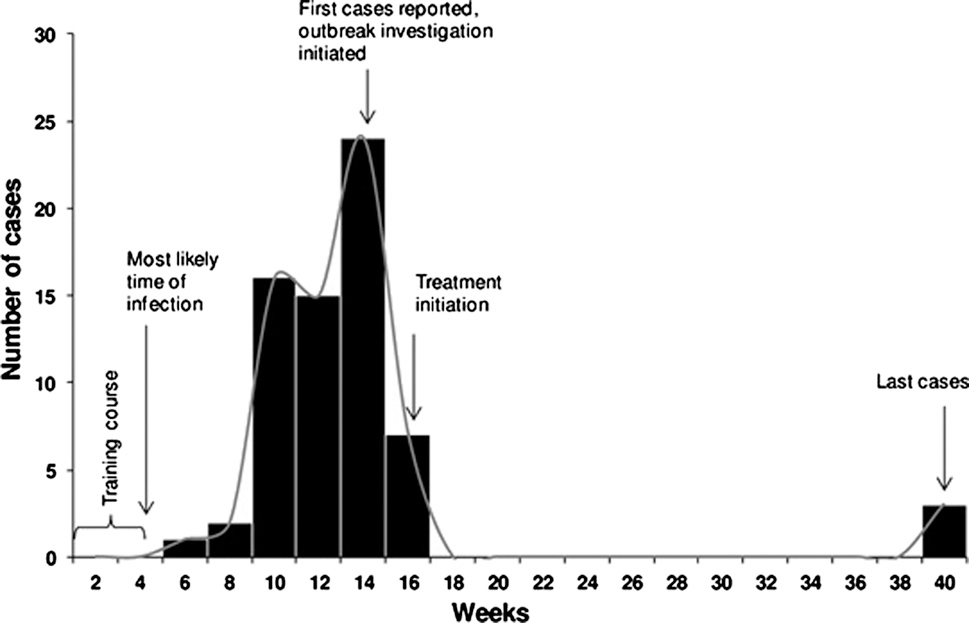
Epidemic curve for confirmed cutaneous leishmaniasis during the outbreak. The curve was constructed based on self-reported dates when individuals first noticed their skin lesions. Eight of the 76 confirmed cases of leishmaniasis (11%) were excluded because of incomplete data.

Physical examination of the 303 personnel revealed 77 (25%) individuals with skin lesions compatible with CL. The potential incubation period, calculated between the time of highest likelihood of exposure (week 4) and the self-report onset of lesions, ranged from 2 to 36 weeks (Figure 2

), with a median of 8 weeks. All but three cases presented within 16 weeks. The epidemic curve had a sharp peak at week 14 with 24 cases, and in addition, another potential but smaller second peak at week 10 (Figure 2). Confirmation of single versus dual peak was not possible with the data available.

All but one (99%) of the 77 military personnel with leishmaniasis-compatible lesions was confirmed as CL cases by laboratory testing, revealing a global attack rate of 25% (76/303). One subject was negative by microscopy, culture, and kDNA PCR and was considered a non CL case (Table 1). Of all the laboratory tests, the kDNA PCR had the highest positivity rate among tested subjects with CL (98.7%; 74/75), followed by direct microscopy (80.6%; 54/67) and culture (52.1%; 25/48). One case was diagnosed solely by microscopy in the absence of additional samples. The real-time PCR assay identified the infecting species in 82.4% (61/74) of the kDNA-positive cases. *L.* (*V.*) *braziliensis* was the predominant species (96.7%), except in two cases infected with *L.* (*V.*) *guyanensis* (Table 1). The causative *Leishmania* species was not determined in 13 (17.6%) of CL cases positive by kDNA PCR but negative by the real-time PCR (Table 1).

The 76 confirmed CL cases had 146 lesions altogether and nearly two-thirds of the cases had a single lesion, although three cases presented with more than 10 lesions (range = 1–11). Lesions occurred predominantly on the legs (57%), and were mainly ulcers (46%) or plaques (34%), although 6.8% of lesions had mixed features (Table 2). No cases of disseminated leishmaniasis were observed, and cases with multiple lesions appeared to result from multiple inoculation sites.

The personnel reported several potential risk factors such as having outdoor activities at dawn/sunset (93.0%), not using insecticide-treated bed nets (83.8%), not wearing long-sleeve clothes (31.7%), and sleeping in rooms with open doors/windows (11.9%). Bivariate analysis revealed that higher CL incidence was associated with being male (RR = 4.37; *P* = 0.009), sleeping in open rooms (RR = 1.83; *P* = 0.014), not wearing long-sleeve clothing (RR = 1.84; *P* = 0.002), and reporting being bitten by sand flies (RR = 2.70; *P* = 0.042) (Table 3). The association between CL and each of these four factors was slightly attenuated after multiple regression adjustment, and reporting sand fly bites was no longer significantly associated with increased CL.

All 76 laboratory-confirmed cases received intravenous treatment with sodium stibogluconate in strict adherence to the Peruvian MoH treatment guidelines but only 31 (41%) achieved clinical cure 8 weeks after treatment was ended. As dictated by the national guidelines, the 45 cases who did not achieve clinical cure after the first course of intravenous sodium stibogluconate initiated a second round of treatment with 20 mg base/kg/day intravenous sodium stibogluconate for 20 days as mandated by MoH norms. The outcome of retreatment will be reported separately.

Military personnel scheduled for survival training in the following year (2011) received pre-training leishmaniasis awareness and prevention briefings, emphasizing the use of insect repellents, strict adherence to long-sleeve clothing, and sleeping in protected areas, issues identified in the risk factor analysis. Training officers were additionally briefed about the risks of leishmaniasis and other vector-borne diseases in the area. One CL case presented in participants of the 2011 survival training courses (0.4%; 1/278) and no cases were observed in 2012 to 2014; this was substantially lower compared with the attack rate in the 2010 outbreak (*P* < 0.001).

## Discussion

We characterized in detail a CL outbreak among military personnel undertaking short-term training in an endemic area of the western Peruvian Amazon Basin. The 25% attack rate was relatively high compared with most previous reports in the Americas (< 1–22%),^10^,^13^–^15^,^22^,^31^–^33^ except for two outbreaks in French Guiana (84% and 91%) with substantially longer exposure periods (80 and 42 days, respectively).^10^ The identification of risk factors strongly associated with CL such as not wearing long-sleeve clothes and sleeping in open rooms, as well as other potential risks allowed the Peruvian Army to provide targeted pre-deployment CL prevention education to trainees and the chain of command. The CL incidence in the following cohort of personnel completing survival training was minimal (0.4%), and while this lower risk cannot be solely attributed to the *Leishmania* education interventions, the use of evidence-based public health probably played an important role in preventing future CL outbreaks. The importance of education in preventing CL among military personnel deployed to endemic settings is well recognized,^34^,^35^ as well as its effect in increasing the adherence to preventive measures.^35^ Nevertheless, the efficacy of such measures for preventing CL outbreaks among military personnel deployed to endemic settings has not been defined. Our data suggest that provision of pre-travel leishmaniasis awareness briefings emphasizing the importance of adherence to personal protective measures may constitute a low-cost and effective measure for preventing CL outbreaks among military personnel deployed to endemic setting, and may be particularly feasible in resource-limited settings.

The high attack rate of CL in this outbreak could be attributed to various factors. First, the Amazon Basin is a highly endemic area for leishmaniasis, and several outbreaks have been previously reported in other countries,^14^–^17^ with the potential coexistence of multiple vectors and reservoirs.^27^ In addition, specific conditions of the training area such as deforestation and recent land use changes due to agriculture could have played a role as well.^36^ Also, personnel lacked acquired immunity to leishmaniasis given that they were all born and raised in non-endemic settings.^10^,^14^–^16^,^22^,^32^,^33^,^37^ Finally, the risk behaviors identified such as the lack of protective clothing, bed nets, or repellents probably played an important role. Other risk factors such as being outdoors at dawn and sunset could have also been implicated in this outbreak, but the small number of CL cases among nonexposed individuals may limit the power of our study to detect such an effect.

Our investigation revealed two potential places of exposure. Military personnel were exposed to the open field for a full day at site B, a rural site moderately endemic for leishmaniasis according to MoH records.^38^ Exposure at site C was longer (4 days), and probably more intense also, as the MoH reports higher leishmaniasis incidence^39^ and military personnel were trained without proper clothing. The high dispersion and late peak of the epidemic curve may be a hint that infection could have taken place in multiple days. Furthermore, the identification of two species of leishmaniasis is in line with the hypothesis of multiple exposure opportunities. However, this hypothesis remains as speculation in the absence of solid, confirmatory evidence and the limited accuracy of self-reported lesion initiation.

kDNA PCR was the diagnostic method that yielded the highest positivity rate for leishmaniasis in this outbreak.^40^ This assay can identify *Leishmania* (*Viannia*) species, but is unable to discriminate species within this subgenus. Our recently developed real-time PCR assay, however, can identify the infecting *Leishmania* species and had a positivity rate of 82% in this outbreak. This real-time PCR assay identified *L*. (*V*.) *braziliensis* as the main etiology of the outbreak in all but two cases that were infected with *L*. (*V*.) *guyanensis.* This finding is important since these two species have been associated with mucosal presentations^41^ and different *Viannia* species have different cure rates after antimonial treatment.^42^ However, *Leishmania* species identification is not routinely conducted in health facilities from endemic settings and the incrimination of an individual species without laboratory confirmation is often difficult if patients have a diverse travel history. Therefore, the use of rapid and accurate species identification in outbreak responses can improve the management of cases, targeting the most effective treatment of the *Leishmania* species and monitoring closely early signs of mucocutaneous lesions.

In this outbreak, the cure rate after sodium stibogluconate treatment reached 41%, which is considerably lower compared with that previously reported in Peru for *L.* (*V.*) *braziliensis* (∼70%).^42^ It is unclear why such a low cure rate was observed in this outbreak. One potential explanation for this phenomenon is the early diagnosis and treatment of cases, as it has been previously reported that early treatment of CL is associated with a higher risk of treatment failure.^42^–^44^ This differential response to treatment may be determined by the profile of the immune response early in the course of infection.^39^,^44^,^45^ The early response to the parasite exhibits a Th2 profile, with low levels of tumor necrosis factor alpha (TNF-α) and interferon gamma (IFN-γ), poor macrophage activation, and parasite replication and persistence.^45^–^47^ In contrast, the Th1 response develops in more advanced phases of infection and is required for parasite eradication. Other potential explanations are the young age of the military population (18–23 years), which was one of the most important predictors of treatment failure in a previous study,^42^ and their lack of acquired immunity from previous exposure to *Leishmania*. Finally, we are currently studying whether *Leishmania* strains from this outbreak have phenotypic resistance to antimonial drugs.

A limitation of the outbreak response was that individual activities and itineraries performed during the training could not be disclosed because they were considered sensitive information. In addition, the lack of knowledge of the geographic distribution of cases, species, and vectors in these remote endemic regions was another limitation, which hindered the accurate identification of the probable time and place of infection during the outbreak.

In summary, this outbreak of CL had a high attack rate after a very short exposure period and emphasizes the high risk for military personnel traveling to endemic settings for operational activities. We identified risk factors associated with higher CL incidence that could be applicable to both military and civilian populations traveling to similar endemic areas. Preventive educational interventions based on these factors probably contributed to reduce CL risk in subsequent years, and highlight the need for evidence-based public health measures in leishmaniasis control.

## Figures and Tables

**Table 1:** Diagnostic results of 75 laboratory-confirmed cases of CL in military personnel who participated in survival training*

Diagnostic method†	*n*/*N* (%)
kDNA PCR	74/75 (98.7)
Direct microscopy	54/67 (80.6)
Culture	25/48 (52.1)
Real-time PCR	61/74 (82.4)
*Leishmania* (*Viannia*) *braziliensis*	59/61 (96.7)
*L.* (*V.*) *guyanensis*	2/61 (3.3)

CL = cutaneous leishmaniasis; kDNA PCR = kinetoplastid DNA-based polymerase chain reaction.

* One case was diagnosed by histopathology and no other test was performed on this subject.

† All diagnostic methods were not applied to all cases. Percentages show the number of positive cases over all cases evaluated by the corresponding method.

**Table 2:** Characteristics of the lesions in 76 confirmed cases of CL among military personnel who participated in survival training

Characteristic	*n*/*N* (%)
Location	
Legs	43/76 (56.6)
Arms	10/76 (13.2)
Trunk	8/76 (10.5)
Face and neck	6/76 (7.9)
Multiple areas*	9/76 (11.8)
Number of lesions per patient	
1	50/76 (65.8)
2	12/76 (15.8)
3	5/76 (6.6)
≥ 4	9/76 (11.8)
Type	
Ulcer	67/146 (45.9)
Plaque	49/146 (33.6)
Scab	16/146 (11.0)
Papule	4/146 (2.7)
Mixed features	10/146 (6.8)

CL = cutaneous leishmaniasis.

* Lesions in more than one location.

**Table 3:** Crude and adjusted* RR (and 95% CI) of CL for potential risk factors among military personnel who participated in survival training

Characteristic	Bivariate analysis			Multiple regression	
	*n*/*N* (%)	RR (95% CI)	*P* value	aRR* (95% CI)	*P* value
Sex					
Female	2/32 (6.3)	1.00	0.009	1.00	0.034
Male	74/271 (27.3)	4.37 (1.13, 16.95)		4.01 (1.11, 14.42)	
Did not wear long-sleeve clothes					
No	41/205 (20.0)	1.00	0.002	1.00	0.005
Yes	35/95 (36.8)	1.84 (1.26, 2.69)		1.71 (1.18, 2.50)	
Outdoor activities at dawn/sunset					
No	3/21 (14.3)	1.00	0.227	1.00	0.223
Yes	73/279 (26.2)	1.83 (0.63, 5.32)		1.98 (0.66, 5.92)	
Did not use insecticide-treated bed nets					
No	8/48 (16.7)	1.00	0.135	1.00	0.210
Yes	67/249 (26.9)	1.61 (0.83, 3.14)		1.53 (0.79, 2.97)	
Slept in open rooms					
No	61/260 (23.5)	1.00	0.014	1.00	0.009
Yes	15/35 (42.9)	1.83 (1.18, 2.84)		1.80 (1.16, 2.79)	
Reported sand fly bites					
No	3/30 (10.0)	1.00	0.042	1.00	0.213
Yes	73/270 (27.0)	2.70 (0.91, 8.05)		2.01 (0.67, 6.01)	

aRR = adjusted risk ratio; CI = confidence interval; CL = cutaneous leishmaniasis; RR = risk ratio.

* Adjusted by sex, not wearing long-sleeve clothes, and sleeping in open rooms.
